# Supplementary material for: Complete continuum of maternity care and associated factors among mothers who gave birth in the last twelve months in Mekane Selam town North-East Ethiopia: A community-based cross-sectional study,2021
Source: PLoS One. 2023 Sep 28;18(9):e0289200. doi: 10.1371/journal.pone.0289200 (PMC10538653; doi:10.1371/journal.pone.0289200)
Supplement: S1 File — (DOCX) [file pone.0289200.s001.docx]

## S2. Annex II: English Version of the Questionnaire

Questionnaires ID__________

Name of town_____________ Kebele_*____________*

**Instruction:** circle the respondents answer from the given alternatives and write respondents answer on the black space for without option.

**Part I: socio-demographic characteristics**

| S/no | Questions | Answers/choices | Skip |
| --- | --- | --- | --- |
| 101 | Age? | ___________ years |  |
| 102 | Religion? | 1. Orthodox 2. Muslim 3. Protestant 4. Catholic 5. Other________ |  |
| 103 | Marital status? | 1. Single 2. Married 3. Divorced 4. Widowed |  |
| 104 | Ethnicity? | 1. Amhara 2. Tigre 3. Oromo 4. Others ________ |  |
| 105 | Educational status? | 1. No education  2. Primary education   1. Secondary education 2. Higher (College and above) |  |
| 106 | Occupation? | 1. House wife 2. Private employee 3. Government employee 4. Merchant 5. Daily laborer 6. Others ________ |  |
| 107 | What is your husband’s educational status? | 1. No education 2. Primary education 3. Secondary education 4. Higher (College and above) |  |
| 108 | What is your husband’s occupation? | 1. Private employee 2. Government employee 3. Merchant 4. Daily laborer 5. Others __________ |  |

**Part II: Maternal health care services related factors**

| 201 | Did you have exposure to mass media to access relevant information on maternal health care services? | 1. Yes 2. No |  |
| --- | --- | --- | --- |
| 202 | How far the health institution from your home in terms of minutes you spent on your foot to reach there? | _______ minutes |  |
| 203 | Did you have autonomy for seeking healthcare services by yourself or together with your husband? | 1.Yes  2. No |  |
| 204 | Did you know danger signs of pregnancy? | 1.Yes  2. No | If No skip to Q-301 |
| 205 | If yes, about which danger signs of pregnancy did you know? | More than one answer is possible  1.Vaginal bleeding  2. Swelling of legs or face  3. Severe headache  4. Blurring of vision |  |

**Part III: Obstetric related factors**

| 301 | Parity? | ___________number |  |
| --- | --- | --- | --- |
| 302 | Did you use Pre pregnancy contraceptive methods in your recent baby? | 1.Yes  2.No |  |
| 303 | Was the recent pregnancy planned? | 1.Yes  2. No |  |
| 304 | Have you Received antenatal care for your recent pregnancy? | 1.Yes  2. No | If No skip to Q-307 |
| 305 | At what weeks have you start first antenatal care visit? | _________ weeks |  |
| 306 | How many times did you receive antenatal care visit? | _______number |  |

| 307 | Where did you give birth your recent baby? | 1. Health facility  2. Home |  |
| --- | --- | --- | --- |
| 308 | By whom did you have assisted during delivery of your recent baby? | 1. Doctor 2. Health officer   3. Nurse  4. Midwife  5. Health extension worker  6. Traditional birth attendant  7. Others________ |  |
| 309 | After you gave birth the recent baby, have you received postnatal care on your health? | 1. Yes  2. No |  |
| 310 | When did you receive your postnatal care? | 1. Within 24 hours  2. 25-48 hours  3. 49-72 hours  4. 73 hours-7 days  5. 8-42 days |  |

**Thank You for Your Participation!!!!!**

**Annex IV: የአማርኛ መጠይቅ (Amharic version)**

የእናትዩዋ መለያ ቁጥር--------------------------------------

ከተማ--------------------------ቀበሌ-----------------

መመሪያ፡ከተዘረዘሩት ምርጫዎች መልስ የያዘዉን ቁጥር በማክበብ እና ምርጫ ለሌላቸዉ ጥያቄዎች ክፍት ቦታዉን በመሙላት ይመለሱ።

**ክፍል1፡- ማህበራዊ ሁኔታዎችን በተመለከተ**

| ተራ ቁጥር | ጥያቄዎች | መልስ | የሚዘለል |
| --- | --- | --- | --- |
| 101 | እድሜ? | _______አመት |  |
| 102 | ሐይማኖት? | 1. ኦርቶዶክስ 2. ሙስሊም 3. ፕሮቴስታንት 4. ካቶሊክ 5. ሌላ ይግለፁ______ |  |
| 103 | የጋብቻ ሁኔታ? | 1. ያላገባች 2. ያገባች 3. የፈታች 4. የሞተባት |  |
| 104 | ብሄር? | 1. አማራ 2. ትግሬ 3. ኦሮሞ 4. ሌላ ይግለፁ______ |  |
| 105 | የትምህርት ደረጃ? | 1. ያልተማረች 2. አንደኛ ደረጃ 3. ሁለተኛ ደረጃ 4. ኮሌጅ እና ከዚያ በላይ |  |
| 106 | የስራ ሁኔታ? | 1. የቤት እመቤት 2. የግል ተቀጣሪ 3. የመንግስት ተቀጣሪ 4. ነጋዴ 5. የቀን ሰራተኛ 6. ሌላ ይግለፁ______ |  |
| 107 | የትዳር ጓደኛዎ የትምህርት ደረጃ ምን ድረስ ነዉ? | 1. ያልተማረ 2. አንደኛ ደረጃ   3. ሁለተኛ ደረጃ  4. ኮሌጅ እና ከዚያ በላይ |  |
| 108 | የትዳር ጓደኛዎ ስራ ምንድን ነው? | 1. የግል ተቀጣሪ 2. የመንግስት ተቀጣሪ 3. ነጋዴ 4. የቀን ሰራተኛ 5. ሌላ ይግለፁ______ |  |

**ክፍል 2፡**- **ከእናቶች የጤና እንክብካቤ አገልግሎት ጋር የተያያዙ ጥያቄዎች**

| 201 | ስለእናቶች የጤና አገልግሎት በተመለከተ ጠቃሚ መረጃ ለማግኘት መገናኛ ብዙሃንን ይጠቀማሉ? | 1. አዎ  2. የለኝም |  |
| --- | --- | --- | --- |
| 202 | ከመኖሪያ ቤትዎ ጤና ተቋማት ለመድረስ በእግርዎ ሰንት ደቂቃ ይወስዳል? | _________ደቂቃ |  |
| 203 | የጤና ክትትል አገልግሎት ለማግኘት በራስዎ ወይም ከባለቤትዎ ጋር በመሆን የመወሰን መብት አለዎት? | 1.አዎ  2.የለም |  |
| 204 | በእርግዝና ጊዜ ሰለሚያጋጥሙ አደገኛ ምልክቶች ያውቃሉ? | 1.አዎ  2.የለም | የለም ካሉ ወደጥያቄ ቁ-301 ይለፉ |
| 205 | መልስዎ አዎ ከሆነ የትኞቹ የእርግዝና አደገኛ ምልክቶችን ያውቃሉ? | ከአንድ በላይ መልስ ይቻላል   1. የደም መፍሰስ 2. የእግር ወይም የፊት ማበጥ 3. ከፍተኛ የሆነ ራስ ምታት 4. የዓይን ብዥታ |  |

**ክፍል 3፡** ከወሊድ ጋር የተያያዙ ጥያቄዎች

| 301 | ስንት ልጅ ወልደዋል? | _____________በቁጥር |  |
| --- | --- | --- | --- |
| 302 | የመጨረሻዉ ልጅዎትን ከማርገዝዎ በፊት የወሊድ መቆጣጠሪያ ተጠቅመው ያውቃሉ? | 1. አዎ  2. የለም |  |
| 303 | የመጨረሻዉ እርግዝና አቅደዉት ነበር ያረገዙት? | 1.አዎ  2. አይደለም |  |
| 304 | በመጨረሻዉ ልጅ እርግዝና ወቅት የቅድመ ወሊድ ክትትል አድርገው ነበር? | 1. አዎ  2. የለም | የለም ከሆነ  ወደ ጥያቄ 307 ይለፉ |
| 305 | ስንተኛዉ ሳምንት ላይ ነበር የመጀመሪያ የቅድመ ወሊድ ክትትል የጀመሩ? | _______ሳምንት |  |
| 306 | ስንት ጊዜ የቅድመ ወሊድ ክትትል አደረጉ? | _______በቁጥር |  |

| 307 | የት ነበር የመጨረሻዉን ልጅ የወለዱት? | 1. ጤና ተቋም  2.. ቤት |  |
| --- | --- | --- | --- |
| 308 | የመጨረሻዉን ልጅ ሲወልዱ ማን ነበር ያዋለደዎት? | 1. ሀኪም  2. ጤና መኮነን  3. ነርሰ  4. ሚድዋይፍ  5. ጤና ኤክስቴንሽን  6. የልምድ አዋላጅ  7. ሌላ ካለ ይግለፁ______ |  |
| 309 | የመጨረሻዉን ልጅ ከወለዱ በኃላ የድህረ ወሊድ ክትትል አገልግሎት አግኝተዋል? | 1. አዎ  2. የለም |  |
| 310 | መቼ ነበር የድህረ ወሊድ ክትትል አገልግሎት ያገኙት? | 1. በ 24 ሰአት ዉስጥ  2. 25-48 ሰአት  3. 49-72 ሰአት  4. 73 ሰአት እስከ 7 ቀን  5. 8-42 ቀን |  |

ስለተሳትፎዎ አመሰግናለሁ!!!
