# Supplementary material for: Complete continuum of maternity care and associated factors among mothers who gave birth in the last twelve months in Mekane Selam town North-East Ethiopia: A community-based cross-sectional study,2021
Source: PLoS One. 2023 Sep 28;18(9):e0289200. doi: 10.1371/journal.pone.0289200 (PMC10538653; doi:10.1371/journal.pone.0289200)
Supplement: Attachment — Submitted filename: Review comment COMC.docx [file pone.0289200.s002.docx]

**Title: Complete continuum of maternity care and associated factors among mothers who gave birth in the last twelve months in Mekane Selam town North-East Ethiopia: A Community-Based Cross-Sectional Study,2021**

The topic is relevant and is justified by the impact of childbirth care models on maternal mortality.

1. This manuscript is properly written and showed interesting findings.
2. The study presents the results of original research.
3. Conclusions are presented in an appropriate fashion and are supported by the data.
4. The article is presented in an intelligible fashion and is written in standard English.
5. The research meets all applicable standards for the ethics of experimentation and research integrity.
6. The authors provided valuable data and it is important to help governments, socialists and gynecologists in decreasing maternal mortality and fetal mortality and related diseases.

However, some questions and suggestions need to be made to further improvement of the manuscript. Authors reported that the list of the study participants was derived from the report of the zonal health office and health extension workers (HEWs). But it is very hard to get this list from health post (HEWs). They usually don't have the updated list, that cannot be true. The authors may miss women who deliver at their home. So, I have a big reservation here, unless you have more justification.

I suggest that discussion section start with a paragraph highlighting the most relevant accomplishments of the research. This section can be improved as follows.

1) Main findings in the first paragraph
2) Comparison with existing literature
3) Strengths and Weaknesses of the study should be clearly highlighted
4) Conclusion
