# Supplementary material for: Complete continuum of maternity care and associated factors among mothers who gave birth in the last twelve months in Mekane Selam town North-East Ethiopia: A community-based cross-sectional study,2021
Source: PLoS One. 2023 Sep 28;18(9):e0289200. doi: 10.1371/journal.pone.0289200 (PMC10538653; doi:10.1371/journal.pone.0289200)
Supplement: Attachment — Submitted filename: PLOS ONE.docx [file pone.0289200.s003.docx]

PLOS ONE

Abstract

- You mentioned that: “Ethiopia remains one of the largest contributors to the global burden of maternal and neonatal deaths due to the low implementation of the complete continuum of maternity care”. If so, I think there is no need of conducting research rather evaluating the implementation and intervene accordingly.
- You use different words to describe complete continuum of maternity care (prevalence, magnitude….), since it is service utilization, it is better if you consider like proportion, coverage, utilization…

Introduction

- Contradicted ideas like: “zero maternal mortality” Versus “reducing maternal mortality to 70 maternal deaths per 100,000 live births”. Which one is correct?
- If the following studies are the same with your study, then why you are interested to repeat it? “According to a study based on EDHS 2016 data, only 9.1% of Ethiopian women completed the continuum of maternity care [11]. The magnitude of the complete continuum of maternity care ranges from 9.7% in Arba Minch Zuria Woreda [12], 12.1 percent in West Gojjam Zone [13], 21.6 percent in Gondar Zuria and Dabat districts [14], and 37.2
  percent in Debre Berhan town, according to various studies conducted in Ethiopian rural districts [15]”. Even from the discussion part: “The study found that the prevalence of a complete continuum of maternity care was 42.4% (95% CI: 37.9%, 47%). The finding of the study was in line with the study done in Motta town and Hulet Eji Enese district 47% [23], Enemay district 45% [24], China 41.5% [25], and Nepal 45.7% [27]”.
- The last paragraph of the introduction (statement of the problem) is not convincing for me having the above studies.

Methods

- Sample size determination- why you use p=12.1% rather 37.2% which could give more than 590 study participants? So your sample is not adequate enough.
- Why multistage? Is that appropriate?
- How did you get the sampling frame for systematic sampling technique?
- “The questionnaire was written in English, then translated into Amharic (the local language) to collect data, and then back to English to ensure consistency”. Did you mean you translated back to English after data collection? How did this ensure the consistency?

Discussion

- Your reasoning is not scientifically sound and it is also poor.
