# Supplementary material for: Complete continuum of maternity care and associated factors among mothers who gave birth in the last twelve months in Mekane Selam town North-East Ethiopia: A community-based cross-sectional study,2021
Source: PLoS One. 2023 Sep 28;18(9):e0289200. doi: 10.1371/journal.pone.0289200 (PMC10538653; doi:10.1371/journal.pone.0289200)
Supplement: Attachment — Submitted filename: Response to Reviewers.docx [file pone.0289200.s004.docx]

**To plos one journal editorial board**

**Title: Complete continuum of maternity care and associated factors among mothers who gave birth in the last twelve months in Mekane Selam town North-East Ethiopia: A Community-Based Cross-Sectional Study, 2021.**

**Manuscript number: PONE-D-22-15235**

**Dear Editor and Reviewers, I sincerely acknowledge your positive and valuable comments, despite the length of time it takes to do so. Below, we have made an effort to respond to each of the points raised by the editor and reviewers.**

**Point by point response for editor**

**Q #1**: Dear Authors, please revise your manuscript in detail and address each and every concerns of the reviewers. Mainly, you are expected to clarify the methods used for this study, the description of result section and discussion section.

**R#1:** Your feedback and suggestions are welcome. We made an effort to improve and clarify the methods, result description, and discussion area specifically based on your suggestions. We made an effort to respond to all comments made by reviewers.

**Point by point response for reviewer one**

**Q #1:** I doubt about the novelty of the title, since there were several published articles. i have also not seen any new variables which is different from other studies. All factors the authors identified were also known from previous studies. What new things you add in this study. Not yet identified.

**R #1:** Thank you for your comments and your view, but in our study setting there is no similar study conducted previously in similar manner. In our opinion, this topic has not been sufficiently examined in our nation; therefore, further research is required to discover the relevant implementation-related elements. Some factors identified in our study have significant association with continuum of maternal care but not identified by the other studies.

Additionally, using complete continuum of maternity care can lower maternal and neonatal mortality and morbidity, but these statistics are still very high in developing nations, including our own, so any study, including the one we conducted, has a role to play in pointing out the issues that need to be resolved and interventions that need to be made.

**Point by point response for reviewer Two**

1. The topic is relevant and is justified by the impact of childbirth care models on maternal mortality.

2. This manuscript is properly written and showed interesting findings.

3. The study presents the results of original research.

4. Conclusions are presented in an appropriate fashion and are supported by the data.

5. The article is presented in an intelligible fashion and is written in Standard English.

6. The research meets all applicable standards for the ethics of experimentation and research integrity.

**R**: **Thank you for your constructive and supportive comments.**

**Q#1:** The authors provided valuable data and it is important to help governments, socialists and gynecologists in decreasing maternal mortality and fetal mortality and related diseases.
However, some questions and suggestions need to be made to further improvement of the manuscript. Authors reported that the list of the study participants was derived from the report of the zonal health office and health extension workers (HEWs). But it is very hard to get this list from health post (HEWs). They usually don't have the updated list, that cannot be true. The authors may miss women who deliver at their home. So, I have a big reservation here, unless you have more justification.

**R#1:** Thank you for your comments and views, as you mentioned, we encountered some challenges in obtaining the lists of women, but by using strong coordination and cross-checking approaches of the data with health extension workers, zonal health office, and the nearby health facilities we obtained the most recent updated list of women's give birth in the last twelve months in our study settings. Additionally, in our nation, the government, the ministry of health, and the regional health offices organize a group of women, one to five, to discuss issues pertaining to maternal health by using this way, we didn't miss women who gave birth at home .

**Q#2**: I suggest that discussion section start with a paragraph highlighting the most relevant accomplishments of the research. This section can be improved as follows.
1) Main findings in the first paragraph
2) Comparisons with existing literature
3) Strengths and Weaknesses of the study should be clearly highlighted
4) Conclusions

**R#2**: we accept your comment and correct based on your recommendation and suggestions.

**Point by point response for reviewer three**

**Q#1:** You mentioned that: “Ethiopia remains one of the largest contributors to the global burden of maternal and neonatal deaths due to the low implementation of the complete continuum of maternity care”. If so, I think there is no need of conducting research rather evaluating the implementation and intervene accordingly.

**R#1**: we accept your comments, but more research is still needed to pinpoint the variables that strongly associated with interventions that span the entire continuum of care. It's also crucial to investigate this issue further to determine how well interventions perform when other studies' findings are put into practice. Without research, we would not be aware of the most recent coverage of the complete continuum of maternity care.

**Q#2:** You use different words to describe complete continuum of maternity care (prevalence, magnitude….), since it is service utilization, it is better if you consider like proportion, coverage, utilization…

**R#2:** we accept your comment and we tried to perform the changes that were required.
**Introduction**
**Q#3:** Contradicted ideas like: “zero maternal mortality” Versus “reducing maternal mortality to 70 maternal deaths per 100,000 live births”. Which one is correct?

**R#3:** we accept and acknowledge your comment, and we have made the necessary changes.

**Q#4:** If the following studies are the same with your study, then why you are interested to repeat it? “According to a study based on EDHS 2016 data, only 9.1% of Ethiopian women completed the continuum of maternity care [11]. The magnitude of the complete continuum of maternity care ranges from 9.7% in Arba Minch Zuria Woreda [12], 12.1 percent in West Gojjam Zone [13], 21.6 percent in Gondar Zuria and Dabat districts [14], and 37.2
percent in Debre Berhan town, according to various studies conducted in Ethiopian rural districts [15]”. Even from the discussion part: “The study found that the prevalence of a complete continuum of maternity care was 42.4% (95% CI: 37.9%, 47%). The finding of the study was in line with the study done in Motta town and Hulet Eji Enese district 47% [23], Enemay district 45% [24], China 41.5% [25], and Nepal 45.7% [27]”.

**R#4:** Thank you for your views and advice, but as you can see from the majority of the studies, the total coverage of the full continuum of care is quite low (less than 50%), so more research is necessary to determine the causes of this low coverage. Additionally, it is not carried out in the same way in our settings.
**Q#5:** The last paragraph of the introduction (statement of the problem) is not convincing for me having the above studies.

**R#5:** Thank you for your feedback and comments. We would like to point out that no studies of this nature have been conducted in our study area, and that the study that has been done on this vast subject is, in our opinion, insufficient.

Methods
**Q#6:** Sample size determination- why you use p=12.1% rather 37.2% which could give more than 590 study participants? So your sample is not adequate enough.

**R#6:** We appreciate your thoughts and viewpoints and we agree with them. We made a mistake when we were looking through the literature to construct the proposal; we added these studies after developing the proposal and gathering the data, which is why we didn't utilize them to determine the sample size. When compared to the populations and sample sizes of other studies, the sample we determined is considerably more substantial than the minimum when we look at the whole population. In general, we want to acknowledge your comment.

**Q#7:** Why multistage? Is that appropriate?

**R#7:** We acknowledge your comments and suggestions. In our studies, the final participants were chosen in stages. Initially, there were seven kebeles in the study area; in the first stage, we chose four kebeles using simple random sampling techniques, and in the second stage, after proportional allocation, we chose study participants using systematic sampling techniques. By taking into account these steps, we were able to minimize errors by using design effects.
**Q#:8**: How did you get the sampling frame for systematic sampling technique?

**R#8:** thank you for your comment. The local health office, which collaborates with the local health institution, and health extension workers who work at the community level provide us with a list of the samples. We can easily structure the sample frame by employing those bodies.

**Q#9:** “The questionnaire was written in English, and then translated into Amharic (the local language) to collect data, and then back to English to ensure consistency”. Did you mean you translated back to English after data collection? How did this ensure the consistency?
Discussion

**R#9:** we accept your comments. English version was translated to Amharic version by two native speakers of Amharic language (one was reproductive health and the other was English language and literatures in professions). Then back translation to English was done by another two individuals who could speak English (again one was from reproductive health and the other from English language and literatures). Individuals involved in translations were those who knew local says for some expressions. Final questionnaire was prepared by involving both groups (translators) after resolving inconsistences via discussion for some meanings and terminologies.
**Q#10:** Your reasoning is not scientifically sound and it is also poor.

**R#10:** We acknowledge your remarks and have made an effort to update the justifications in light of science.

**Point by point response for reviewer four**

Title: Complete continuum of maternity care and associated factors among mothers who gave birth in the last twelve months in Mekane Selam town North-East Ethiopia: A Community-Based Cross-Sectional Study, 2021
Comments:
1. Abstract section:
**Q#1**: Method – in method section you have to put the original sample and it was 479, and then in the result section you have to put the response rate.

**R#1:** we accept the comment and we tried to correct based on suggestions.
**Q#2:** The conclusion is not informative, therefore it need revision.

**R#2:** thank you for your comments and suggestions. We tried to revise accordingly.
2. Introduction:
**Q#3:** Please revise paragraph three. The SDG goal is not exactly to make the maternal mortality 0, instead
“The SDG focus on the reduction of the global maternal mortality ratio to at least <70 per 100 000 live births or no country has no more than 140 maternal mortality ratio, reducing neonatal deaths to 12 per 1000 live births, and under-five deaths to <25 per 1000 live births through eliminating preventable maternal, neonatal and child deaths by the year 2030”

**R#3:** Your comments and suggestions are welcome and acknowledged. Accordingly, we corrected.

**Q#4:** Paragraph four: why you used EDHS 2016? Please use current data at least from EDHS 2019

**R#4:** We acknowledge your feedback and suggestions, but since we used EDHS 2016 for our research, the 2019 EDHS study only presented the outcomes of the ANC follow-up, labor and delivery, and PNC assessments separately; it did not present the complete continuum of care as was done in EDHS 2016. But we used 2019 Ethiopian Mini Demographic and Health Survey for comparisons of each components of complete continuum of care.

**Q#5:** Again in paragraph four rewrite the following sentence - The magnitude of the complete continuum of maternity care ranges from 9.7% in Arba Minch Zuria Woreda [12], 12.1 percent in West Gojjam Zone [13], 21.6 percent in Gondar Zuria and Dabat districts [14], and 37.2 percent in Debre Berhan town, according to various studies conducted in Ethiopian rural districts [15] (please write your article as researcher)
-Please put % instead of the word percent after number (s)
-In the last paragraph, what do you mean the term ‘volume?’

**R#5:** we acknowledge your comment and we rewrite the paragraph accordingly.
3. Methods:
**Q #6**: In the study design and sitting you included the expected population of the town, please indicate for which year ___, and then put the reference .

**R#6**: we accept your comment. At the time this research was done, town administrative entities provided us with the population total, which we sought to modify based on your suggestions.

**Q#7:** your sampling procedure is not clear. How many kebeles existed in the town, and how did you select among them? Make it clear and try to show it in figure.

**R#7:** Accept your comment and we stated clearly.
**Q#8:** In sampling procedure I have a question – Which sampling technique is best for community based study? You have a sampling frame as you stated, so why did you used systematic sampling instead of simple random sampling technique?

**R#8:** Thank you for your thoughtful comments and suggestions. Our study was conducted at the community level, and the systematic sampling method has the advantage of allowing us to collect data from house to house within a predetermined window of time while working with a limited financial and human resources in scattered villages over a large area.

**Q#9:** In the result section you stated the response rate was 97.9%, is it because of absent, if so what did you do? Or because of other issue. Please clarify it.

**R#9:** We accepted your comment. We have a 2.1% non-response rate for a variety of reasons, including incomplete questionnaire responses, participants who felt compelled to stop after giving formal consent, and others. Some study participants missed the first day of data collection, but we made an effort to gather them the next day and up until the completion of data collection.

**Q#10:** In operational definitions –You listed only four danger signs of pregnancy and you classified the women as knowledgeable on danger signs of pregnancy if she mentioned two or more danger signs you listed. Is swelling of legs is a danger signs of pregnancy? There are more danger signs of pregnancy, which you didn’t included in the list (it was best if you include all of the danger signs of pregnancy and assessed based on mean value)

**R#10:** We acknowledge and accept your feedback. Although there are other danger signs, as you pointed out, we chose these operational definitions after looking different literatures. If a woman recognizes two or more danger indicators, it indicates that she had knowledge and awareness about the danger signs. In general, we accept your feedback and suggestions and agree with what you say.
**Q#11:** Data collection tools and quality measurements –please avoid redundancy (see it)

**R#11:** We have taken note of your suggestions and made the necessary adjustments.

**Q#12:** Data processing –how many outcome variable did you have, make it ‘outcome variable’

**R#12:** we accept your comment. We have one outcome variable and we were write accordingly.

**Q#13:** Ethical consideration – did you obtained both verbal and written consent? Choose one and include IRB number

**R#13:** We acknowledge your comment and have made the necessary corrections.
4. Results:g
**Q#14:** Avoid the term ‘respondent’ in your document – replace with the term ‘Women’And rewrite your results, b/c some of them didn’t give sense e.g. more than half of the 242 people who responded (51.6%) were between the ages of 25 and 34.

**R#14:** We accept your comment and have made the necessary corrections.

**Q#15:** In the table if the exact number is not 469, put in bracket after the variable. e.g., 337 of women have PNC visits, then you have to put like this ‘timing of PNC visits (n=337)

**R#15:** thank you for your comment. We made correction accordingly.
5. Discussion:
**Q# 16:** See the 1st paragraph –replace the term study with studies

**R#16:** We accept your comment and we replaced it.
**Q#17:** Paragraph two - Discuss your result with more current information –use current EDHS data

**R#17:** We acknowledge your feedback and suggestions, but since we used EDHS 2016 for our research, the 2019 EDHS study only presented the outcomes of the ANC follow-up, labor and delivery, and PNC assessments separately; it did not present the complete continuum of care as was done in EDHS 2016.

**Q# 18:** Paragraph 3 – did you include 6 weeks or 48 hrs to say completed continuum care of maternity? The explanation must be evidence based

**R#18:** Thank you for your comments. In our study we used the 1^st^ 48hrs because the majority of complications occur in the 1^st^ two days of child birth and also we employed EDHS as a reference in our study.
6. Conclusion:
**Q#19:** The conclusion have to be based on your finding

**R#19:** We accept your comment and we tried to made correction accordingly.
**Q#20:** The recommendation should be based on the gab you identified, and achievable, and clear (please revise it)

**R#20:** we accept your comment and we tried to modify accordingly.

**Thank you very much Abrham Debeb**

**Email: abrishdebeb86@gmail.com**

**Phone No. : +251 910318281**
